# Molecular monitoring of viral infections in immunocompromised patients in a large university hospital in Italy: reflections after thirteen years of real-life activity

**DOI:** 10.1007/s10096-024-04812-z

**Published:** 2024-03-22

**Authors:** Lilia Cinti, Piergiorgio Roberto, Matteo Rossi, Anna Napoli, Gianluca Russo, Anna Paola Iori, Giuseppe Gentile, Maria Augurusa, Corrado Girmenia, Guido Antonelli, Aurelia Gaeta

**Affiliations:** 1 Laboratory of Microbiology and Virology, Department of Molecular Medicine, Sapienza University of Rome, Viale Regina Elena, Roma, 324-00161 Italy; 2 Department of Public Health and Infectious Diseases, Sapienza University of Rome, Roma, Italy; 3 Hematology, Department of Hematology, Oncology and Dermatology, AOU Policlinico Umberto I, Sapienza University of Rome, Roma, Italy; 4 Department of Translational and Precision Medicine, Sapienza University Rome, Roma, Italy; 5 University Hospital “Policlinico Umberto I”, Rome, Italy

**Keywords:** Epidemiology, Letermovir, COVID-19, Cytomegalovirus (CMV), Epstein-Barr Virus (EBV), Polyomavirus (JCV, BKV)

## Abstract

**Purpose:**

This study aimed to investigate the prevalence and viral reactivations of clinical interest in the immunocompromised patient with particular focus on hematologic and solid organ transplant recipients.

**Methods:**

Molecular screening data of CMV, EBV, JCV and BKV from 2011 to 2023 were analyzed. This extensive time span allowed the access to more than 100,000 samples from over 20,000 patients treated at Policlinico Umberto I. It was possible to temporally investigate patient attendance patterns, average age distribution, seasonality of infections, and positivity rates of the analyzed viruses.

**Results:**

Between 2019 and 2022 a significant reduction in organ transplants performed and in the positive molecular detection of EBV, JCV and BKV was observed. Additionally, there has been a noteworthy decrease in CMV reactivations, with a reduction of up to 50% starting in 2019. A remarkable reduction of 39% in the rate of CMV viral reactivation has been also achieved in SOT between 2016 and 2023.

**Conclusion:**

The years following 2019 were profoundly impacted by the COVID-19 pandemic era. This period resulted in a substantial reduction in healthcare services and hospital visits. Furthermore, the introduction of the drug Letermovir in Italy in 2019 demonstrated remarkable efficacy, evidenced by a reduction in CMV reactivations. Additionally, the adoption of a novel clinical approach centered on personalized therapy facilitated improved management of immunocompromised patients.

**Supplementary Information:**

The online version contains supplementary material available at 10.1007/s10096-024-04812-z.

## Introduction

The evolution of diagnostic methods has facilitated enhanced analytical sensitivity through the utilization of molecular techniques. To date, molecular investigation of the viral load has assumed over the years a crucial role in ensuring precise diagnosis of infections. Within the cohort of vulnerable patients, such as those undergoing solid organ transplantation (SOT) or hematopoietic stem cell transplantation (HSCT), primary viral infections or reactivations bear significant implications for patient survival [1].

Consequently, careful monitoring of specific viruses is included in clinical protocols for immunocompromised conditions. Particular emphasis is placed on two *Herpesviridae* family members: Cytomegalovirus (CMV) and Epstein-Barr Virus (EBV), recognized as principal determinants of systemic infectious complications other than graft rejection, therapeutic failure or post-transplant proliferative disorders [2–4]. Equally important is the surveillance of JCV and BKV viruses belonging to the *Polyomaviridae* family. Indeed, in cases of immunodeficiency, JCV has the potential to disseminate within the central nervous system (CNS), culminating in progressive multifocal leukoencephalopathy (PML), a severe demyelinating disorder [5]. Conversely, BKV reactivation represents the predominant cause of transplant loss in kidney transplant recipients [6].

The accumulation of a substantial number of patients and a vast amount of data over the years in a large university hospital holds the potential to offer novel insights into the understanding of the epidemiological dynamics of these viruses.

In this study, viral molecular screening data produced by the Sapienza University Hospital “Policlinico Umberto I” (SU-PUI) in Rome from the beginning of 2011 to the end of 2023 were analyzed. One of the aims of afore effort is to observe, through statistical analysis, the patterns of infections and spread of the above-mentioned viruses in various hospital department.

Particular emphasis will be paid to the aforementioned issues in the period of the COVID-19 pandemic compared to the previous period, i.e. before 2020. It is indeed worth noting that the COVID-19 pandemic could have had an important impact specifically in immunocompromised patients since the immune status of subjects is one of the determinants of the severity of the course of infection [7, 8]. It follows that the study of the specific viruses included in clinical protocols in the subjects covered by this study in the above period is of particular relevance.

## Materials and methods

### Population study

A retrospective analysis was conducted on 113,097 samples collected from 20,584 patients who received medical care at SU-PUI in Rome between January 1st, 2011, and December 31st, 2023. Among these patients, 54% are male, whereas the remaining 46% are female, with a mean age of 39 years (IQR: 11–59).

The study focuses on the analysis of molecular test requests for CMV, EBV, JCV and BKV viruses, including plasma (92%) urine (3%), cerebrospinal fluid (CSF) (4.5%) and bronchoalveolar lavage (BAL) (0.5%). Sample types with insufficient numerical representation for statistical analysis were excluded. Moreover, the collected samples were categorized into five distinct groups, based on the patients’ respective wards of origin. Specifically, the “Hematology” (HEM) category included samples of patients from the outpatient clinic, hematology emergency room, as well as those hospitalized for hematologic diseases. In particular HEM category comprises patients with a median age of 50 (IQR: 32–64) years, undergoing allogeneic stem cell transplantation (SCT), autologous SCT and among hematologic non-HSCT patients (patients with acute or chronic lymphoid malignancies, patients receiving T-cell suppressive therapy, patients with Non Hodgkin lymphoma receiving rituximab, patients receiving intensive chemotherapy for different types of acute leukemia) most at risk of viral reactivation [9].

The “solid organ transplant” (SOT) category comprises samples from patients who have undergone kidney, liver, or lung transplantation. In general, SOT recipients involved in this study have a median age of 52 (IQR: 42–61) years, affected by more than one co-morbidity, assuming anti-rejection immunosuppressive regimens mainly based on calcineurin inhibitors (i.e. tacrolimus or cyclosporine) with or without low-dose of corticosteroids, and possibly with an additional immunosuppressive drug (i.e. mycophenolic acid, mTOR inhibitors) according to the transplanted graft and patient’s clinical conditions. As far as diagnostic prescriptions, usually the clinicians used antiviral prophylaxis and, especially for CMV, a pre-emptive approach (i.e. starting therapy or prophylaxis according to the level of viremia and characteristics of the individual patient) [10].

The “medical clinic” (MED) category referred to samples from diverse array of departments, including cardiology, nephrology, gastroenterology, neurology, oncology, gynecology, and obstetrics. The “other” (OTR) category includes specimens from patients admitted to other types of wards for those, such as surgical, infectious diseases, cystic fibrosis, neonatology and hospital emergency rooms cases. Furthermore, the Intensive Care Unit (ICU) category encompasses several specialized units, such as those dedicated to transplants, COVID-19 patients, neonatal care, as well as anesthesia and reanimation wards.

### Molecular analysis

Molecular analysis to detect CMV, EBV, BKV and JCV DNA in clinical samples was conducted at the Virology Laboratory of the Umberto I Polyclinic in Rome using two distinct methods sequentially. From January 2011 to August 2019, DNA extraction step was carried out using the NucliSENS easyMAG instrument (bioMérieux S.p.A., Bagno a Ripoli, Italy), in accordance with the manufacturer’s instructions. The extracted samples were then amplificated using commercially available kits (ELITechGroup SpA) and analyzed using ABI Prism 7300 Real-Time PCR System (Applied Biosystems). The system’s detection linear range spans from 250 to 2.5 × 10^6^ genomic copies/ml. Starting from September 2019, viral load detection was performed utilizing a new automated instrument for nucleic acid extraction and PCR set-up: the ELITe GALAXY (ELITechGroup SpA). The amplification of the viral DNA was carried out through the ELITe CMV/EBV/JCV/BKV MGB^®^ Kits (ELITechGroup SpA), which are designed with specialized primers and probes tailored for identifying the exon 4 region of CMV’s Major Immediate Early Antigen (MIEA) gene, the EBNA-1 gene of EBV, and the Large T Antigen gene of both BKV and JCV. The amplification reaction was then conducted using the ABI Prism 7300 Real-Time PCR System thermal cycler (Applied Biosystems) with the following conditions: an initial decontamination step at 50 °C for 2 min, followed by an initial denaturation step at 94 °C for 2 min. The amplification process comprised 45 cycles, each consisting of denaturation at 94 °C for 10 s, annealing at 60 °C for 30 s, and extension at 72 °C for 20 s. A final step was performed at 80 °C for 15 s to ensure the completion of the reaction. The ELITe CMV/EBV/JCV/BKV MGB^®^ Kits feature a detection linear range spanning from 455 genomic copies/ml to 2.5 × 10^6^ copies/ml for CMV DNA, from 350 to 3.5 × 10^6^ copies/ml for EBV and BKV and from 700 to 3.5 × 10^6^ for JCV.

### Statistical analysis

Data manipulation and visualization were executed in RStudio https://www.rstudio.com/. Null hypothesis testing

was performed using either Mann–Whitney–Wilcoxon or the non-parametric chi-square test methods.

## Results

The study examines data related to multiple viral agents, which have been analyzed separately for the sake of methodological simplicity.

### CMV

**Fig. 1:**
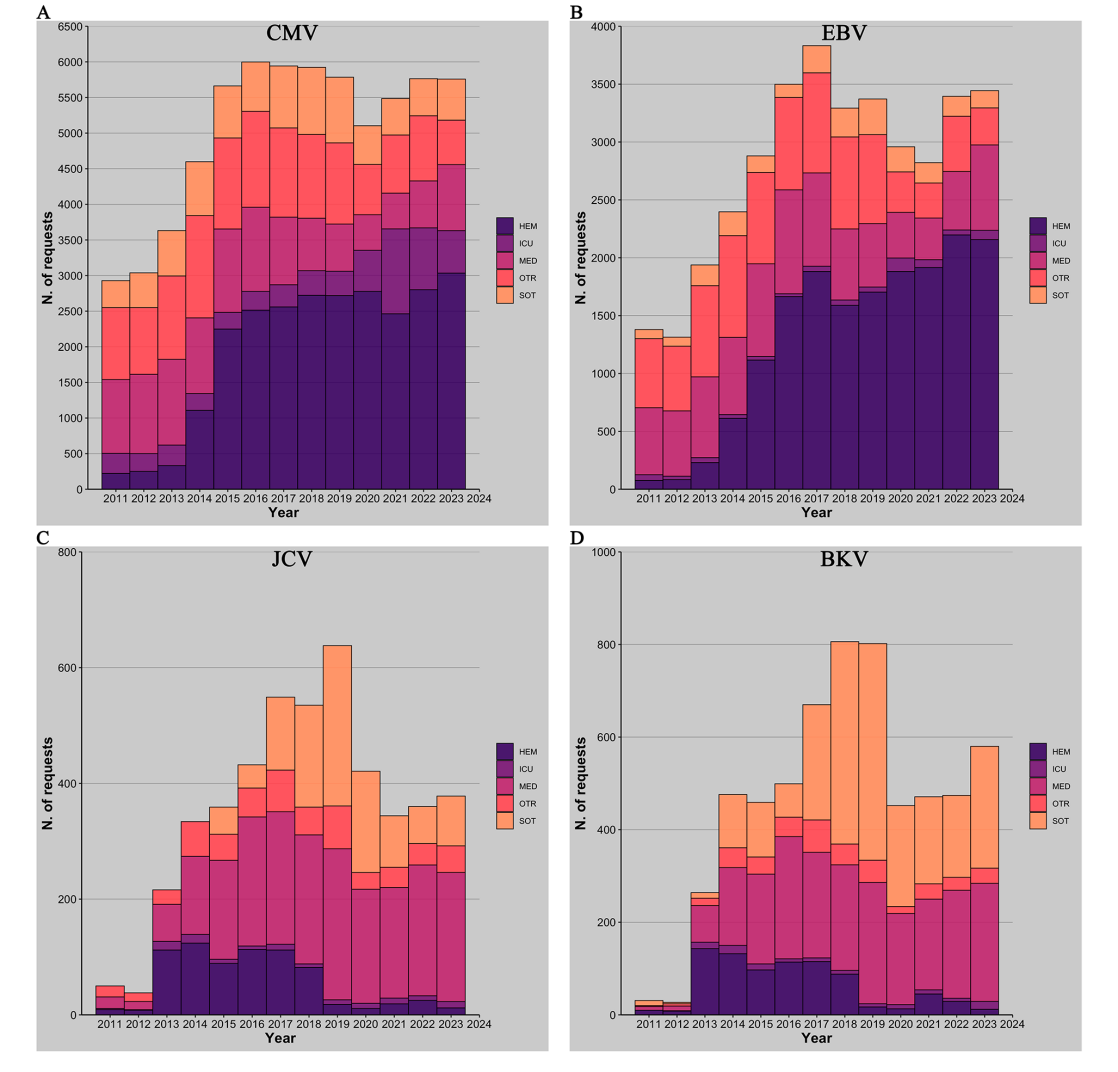
Number of annual requests for CMV (**A**), EBV (**B**), JCV (**C**) and BKV (**D**) viral load research. In the different colors, the categories including the various departments are represented

**Fig. 2:**
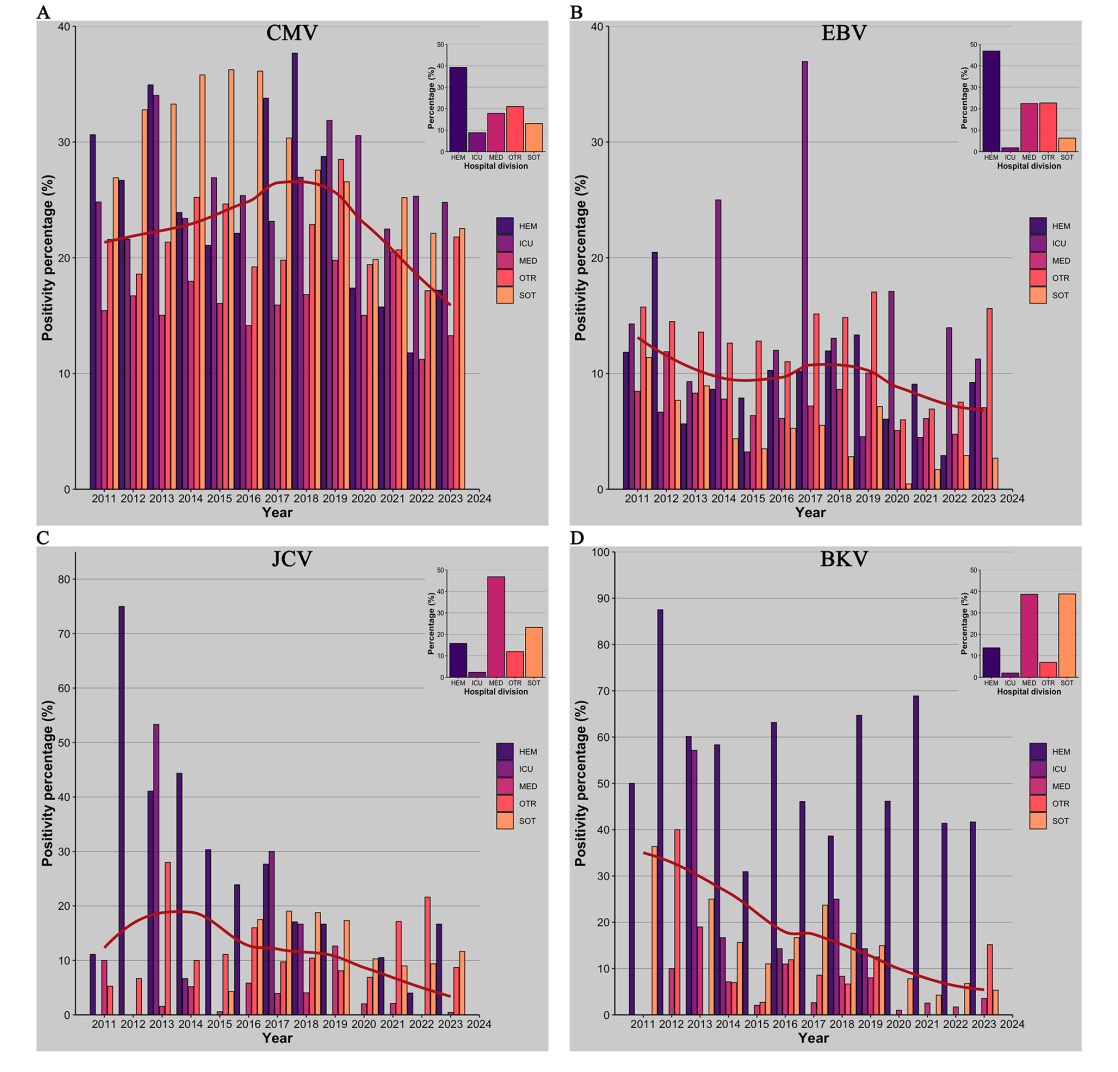
Percentage of annual positivity to CMV (**A**), EBV (**B**), JCV (**C**) and BKV (**D**) relative to total number of samples. A loess regression line is depicted in red. In the different colors, the categories including the various departments are represented. The insets show, as a percentage, the origin of the samples divided into the categories of departments

Firstly, an analysis was conducted to evaluate the consistency of requests for molecular detection of CMV viral load in patients admitted to Policlinico Umberto I of Rome between 2011 and 2023 for a total of 65,906 requests. Among these 94% were related to blood samples (plasma), followed by 3% relative to urine, 2.5% to CSF and 0.5% to BAL samples. The data relatives to the number of requests indicated a notable increase over the years except for a slight decline in 2020 (Fig. 1A). The positivity rate of samples examined for CMV molecular testing was also analyzed. Notably, the positivity percentage exhibited a surge, starting at 21% in 2011, reaching a peak of 29% in 2018 and then considerably declined to 18% in 2023 (*p* < 0.005) (Fig. 2A). The analysis of the requests was subsequently expanded by dividing the samples according to their hospital division of origin grouping them in the following classes previously described: HEM, ICU, MED, SOT, and OTR. The HEM category, representing 38% of total requests, experienced a sudden annual increase from 2011 to 2015 (*p* < 0.005), stabilizing around 2750 in subsequent years, with a slight decline noted in 2021 to 2476 requests (Fig. 1A). A dramatic decline was also observed for the positivity percentage, decreasing from 37.6% in 2018 to 17.3% in 2023 (*p* < 0.005) (Fig. 2A). The ICU group exhibit a significant increase (*p* < 0.005) in the number of requests from 2020 to 2022, reaching a peak of 1192 requests in 2021. The number of requests from MED category remained relatively constant until 2016 when a decline in the number begins, reaching its lowest value in 2020 with 500 requests (Fig. 1A). The number of requests from SOT group show a consistent upward trend culminating in 2019. Between 2020 and 2022 this value was almost halved with an average of 527 requests (Fig. 1A). There was also a reduction in the percentage of positive results from 36% in 2016 to around 20% in the following years (*p* < 0.005) (Fig. 2A). The requests that fall under the OTR category experienced a peak in 2019 then the number decrease in 2020.

**Fig. 3:**
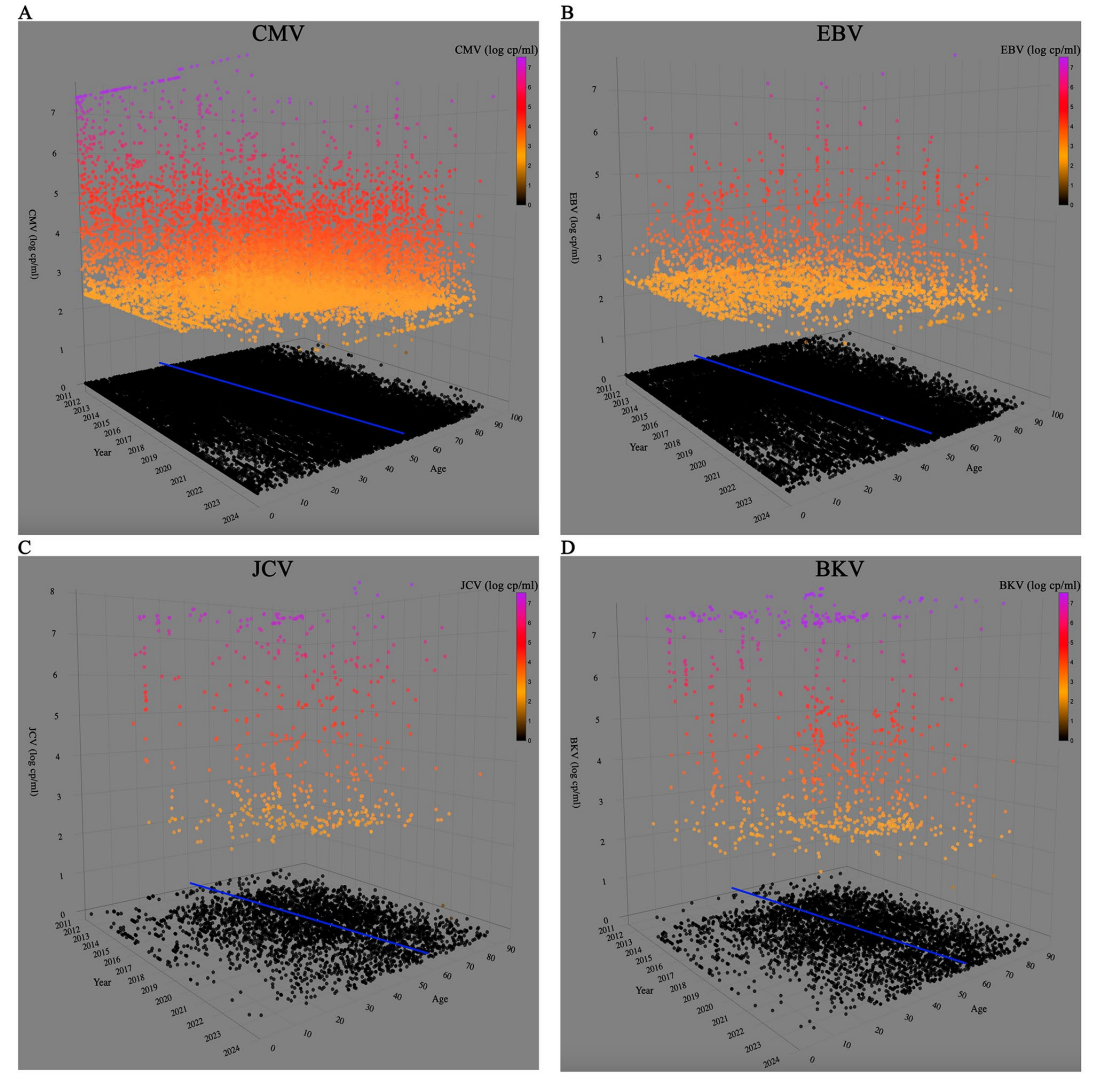
A three-dimensional scatter plot is represented for CMV (**A**), EBV (**B**), JCV (**C**) and BKV (**D**). Each dot represents a sample analyzed in the time frame. Negative samples identified through molecular search for the respective virus are displayed in black. The color scale ranging from yellow to purple represents the variation in the number of genomic copies per ml. Additionally, a blue regression line represents the mean age of the patients

The 3D scatter plot (Fig. 3A) shows the analysis of the number of samples divided by age and CMV DNA load (genomic copies/ml). Between 2011 and 2023, the average age of patients undergoing CMV molecular testing has increased by approximately 20 years, rising from 30 to 50. Two distinct peaks in the CMV copy number are discernible near two specific age groups: between 0 and 1 year, and between 50 and 70 years.

**Fig. 4:**
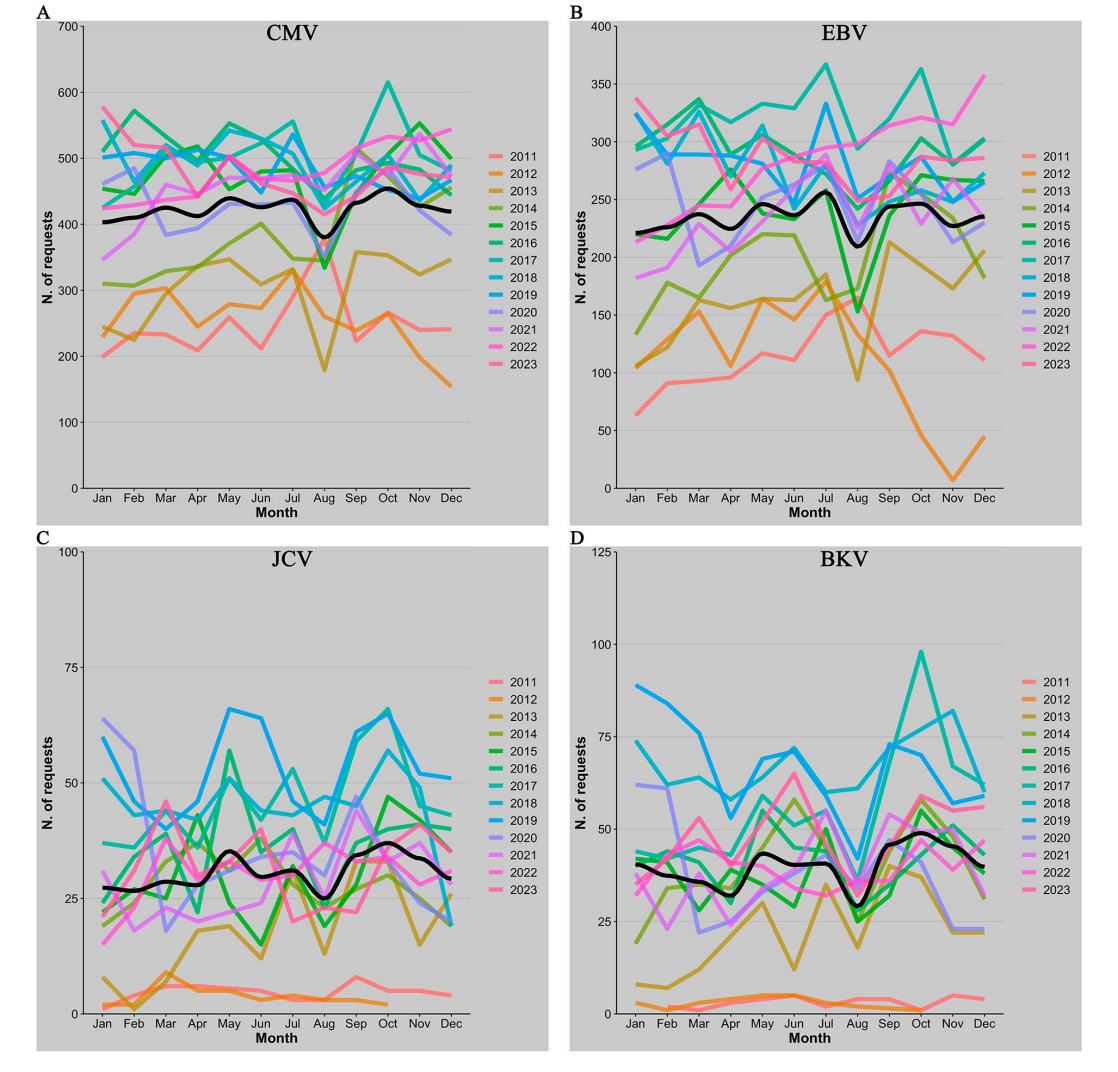
Number of requests for each month of the year for the different viruses: CMV (**A**), EBV (**B**), JCV (**C**) and BKV (**D**). Each line corresponds to a calendar year while the loess regression line is shown in black

Finally, a thorough investigation was conducted into the number of requests and the percentage of positivity in relation to the months of each year spanning from 2011 to 2023 (Fig. 4 A). The regression line in the image indicates a peak in the positivity rate and a decline in the number of requests both during the month of August.

**Fig. 5:**
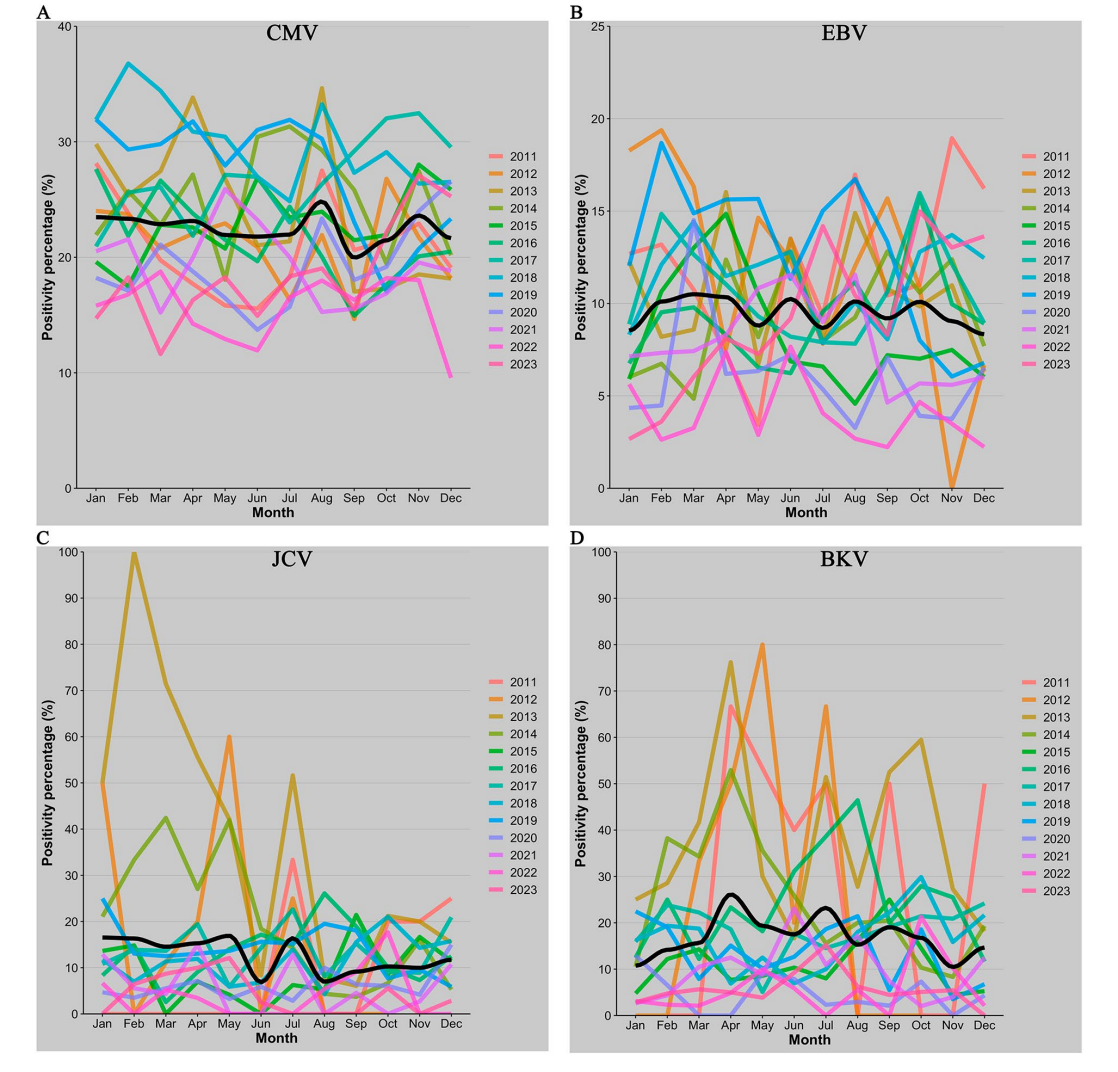
Seasonality expressed as the percentage of positive cases relative to the number of requests for CMV (**A**), EBV (**B**), JCV (**C**) and BKV (**D**). Each line corresponds to a calendar year while the loess regression line is shown in black

### EBV

Between 2011 and 2023, 36,526 requests were processed for the molecular detection of EBV genome. Among these 96% were related to blood samples (plasma), followed by 3.6% to CSF and 0.4% to BAL samples. A growth in the number of requests is evidenced between 2011 and 2017 then decreasing until 2021. The average percentage of positive results was around 11% from 2011 to 2019 and almost halved in the period from 2020 to 2023 (5.9%) (*p* < 0.005) (Figs. 1B and 2B). The HEM group prevailed as the most represented division, accounting for 45.2% of all requests. Requests from this group exhibit a general upward trend, peaking in 2022 with 2197 requests. After 2019, the number of requests of OTR category experienced a rapid decline with its lowest value of 303 requests in 2021(*p* < 0.005). MED macro-category number of requests show the lowest point in 2021 corresponding to 360 requests. ICU group exhibits stability in the number of annual requests except for a single peak of 117 requests in 2021. An examination of the annual EBV positivity rates across different departments reveals a marked decline in the HEM, SOT, and OTR categories, limited to 2020 and 2022.

Similar to CMV, the average age of patients increases over time from 27 years in 2011 to 48 years in 2023 (Fig. 3B).

Seasonal trends in EBV DNA detection do not reveal significant variations in the percentage of positivity at specific times of the year but a slight decrease in the number of requests is observed in the months of August (Figs. 4B and 5B).

### JCV

Over the 13 years a total of 4654 samples were analyzed, comprising 36% plasma samples, 17% urine, and 47% CSF. Since the different sample types contribute comparably to the total, the positivity rates for each sample type were investigated. The highest positivity rate is observed in urine samples, accounting for 36.6% of the total (Table S1). Overall, the trend of requests and the respective positivity rate declined between 2020 and 2023. The years 2011 and 2012 have been deemed insignificant for analysis owing to their inadequately sized samples lacking statistical relevance. Upon stratifying by departments, MED group accounts for the majority of requests at over 47% (Fig. 1 C). HEM cohort exhibited a decline (*p* < 0.005) in number of requests from 2019 to 2023 (Fig. 1C). SOT group displayed a decrease both in number of requests and positivity percentage from 2019 (*p* < 0.005). ICU group had a small number of requests, rendering statistical analysis impractical. Still, the average age of patients increases by 20 years, from 38 to 58 from 2011 to 2023 (Fig. 3C).

As already mentioned for CMV and EBV there is no seasonality, the only observation is a depression in the number of requests in August (Fig. 4 C).

### BKV

Finally, for BKV, molecular research analysis was conducted on a total of 6011 samples, distributed as follows: 48.4% plasma, 17.6% urine, and 34% CSF. Similar to JCV, the highest detection rate was observed in urine, with a 40% positivity rate (Table S1). The requesting departments with the highest representation are MED (39%) and SOT (39%) (Figs. 1D and 2D). The trend observed over the past 13 years indicates a decline both in the number of requests and in the positivity rate between 2020 and 2023 (*p* < 0.005). The decrease in the number of requests is particularly noticeable in the SOT and HEM categories. However, it is important to note that due to the limited number of samples, the temporal analysis divided by departments does not allow for statistical significance in describing the trend of the positivity rate.

Similar to other viruses, there is no apparent seasonality observed for BKV. A decrease in the number of requests is noticeable only in the month of August (Figs. 4D and 5D).

## Discussion

CMV, EBV, BKV and JCV viruses are constantly circulating in the population resulting in self-limiting infections and in most cases establishing a lifelong latent infection. In immunocompromised patients, however, the primary infection or the reactivation of these viruses can progress to symptomatic disease representing the cause of serious complications that in some cases put the patients’ lives at risk [11, 12].

This retrospective study provides an overview on CMV, EBV, JCV, and BKV DNA analysis over the past 13 years in patients admitted to SU-PUI in Rome. The extreme copiousness of data from Europe’s largest hospital and the extensive timeframe considered allowed for highly interesting and significant analyses, to be confined not only to the Roman hospital context but could also faithfully reflect what has occurred on a larger scale in recent years.

Data analysis highlighted a general marked increase in the number of requests from 2011 to 2015. This can be attributed to the assimilation at the Virology laboratory, during that time period, of the conspicuous number of requests previously conducted independently in other decentralized laboratories. Across the entire timeframe, a deviation from the prevailing trend is evidenced in the years 2020–2022, coinciding with the SARS-CoV-2 pandemic. During this period, there was a dramatic fall in requests, particularly in some departments such as SOT, MED, and OTR. Among SOT patients, there was a substantial reduction in the overall number of requests for all examined viruses’ molecular tests. In the case of JCV and BKV, which are frequently analyzed in those patients, the number of requests decreased by up to 52% and 68% respectively (*p* < 0.005). This trend can be traced back to the concomitant decline in patients and transplants performed during the pandemic years [13]. At the same time there is a drastic increase in CMV requirements from ICUs with a 345% increase in 2021 (*p* < 0.005), considering that CMV reactivation is a frequent complication in intensive care units [14]. Similarly, for EBV a closely requests increase was evidenced, corresponding to 260% in 2020 (*p* < 0.005), while for JCV and BKV it is impossible to note significant changes during these years, due to the limited number of annual requests. Furthermore, a general decline in positivity rates was detected during the same years. It is plausible that the preventive measures implemented during the pandemic years, such as the use of facial masks and social distancing, may have had negative impact on the spread of other viral agents than SARS-CoV-2. This has already been observed for viruses in which airborne represent the major route of transmission as in the case of Respiratory Syncytial and Influenza viruses [15, 16]. In the present study a substantial reduction of approximately 50% in the positivity rate of EBV, JCV, and BKV was observed during the pandemic years. Exacerbated concept is the significant decline in BKV by 81% in MED departments (*p* < 0.05), and by 94% for EBV in SOTs (*p* < 0.005).

Concerning CMV, as shown in Fig. 2A, the positivity rate is strongly influenced by the HEM and SOT categories. It exhibits a pre-pandemic decline, unlike the patterns observed for other viruses. This implies that the decline in positivity rates is not solely attributable to COVID-19 pandemic. It is tempting to speculate that the turning point in 2018 observed in HEM category could be ascribed to the introduction of Letermovir, a prophylactic drug against CMV reactivation in hematology patients, which was introduced in Italy in 2019 [17]. This event, 5 years later, seems to have driven down the rate of CMV positivity in hematological patients of around 50% (*p* < 0.005). It is essential to specify that this drug is exclusively administered to hematological patients who have undergone allogeneic stem cell transplantation. Nevertheless, it is noteworthy that this category still constitutes over 85% of the hematological patients analyzed, ensuring that it does not significantly impact the statistical analysis. A similar trend can be observed in the SOT category, with a 39% decrease from 2016 to 2023 (*p* < 0.005). Unlike the HEM category, this decrease cannot be attributed to the introduction of novel drugs or therapies, but rather to the improved plasticity of existing ones. Therefore, the focus in recent years to create personalized therapies [18] tailored to the individual patient has likely led to enhanced control over CMV reactivation by effectively managing their immunosuppression.

Although seasonality was expected for EBV with a peak in winter [19], it was not found in any of the viral agents analyzed. Generally, there is a decrease in the number of requests in August, which in CMV in particular coincides with an increase in the positivity rate. This could be explained by a lower influx of patients in this month, resulting in a higher proportion of analysis requests targeting those patients requiring monitoring. An interesting point regards the general increase in the average age of patients undergoing molecular screening. This trend can be partly explained by the rising number of requests from hematology and SOT departments, whose patient population tends to be older and by a marked decrease in the number of requests for patients under 25 years old from 2019 to 2023.

In conclusion, it can be inferred that the COVID-19 pandemic has resulted in a significant reduction in healthcare services and hospital visits. However, the preventive measures adopted in this period have demonstrated an impressive effectiveness, with transmission rates decreasing by up to 94%, notably, at least for immunocompromised patients. In addition, significant changes have been brought by the introduction of novel drugs and healthcare strategies.

Given these unpredictable events, constructing a reliable probabilistic model to predict future trends remains challenging. Nonetheless, some observations can be made: the number of requests shows signs of rebound starting from 2022, suggesting a potential recovery to pre-COVID-19 levels or even surpassing the pre-pandemic upward trend. Additionally, the positivity rates of EBV, JCV, and BKV are expected to gradually return to pre-2020 levels with the gradual easing of restrictions and safety measures. The scenario is different for hematology patients, as the introduction of Letermovir has shown pandemic-independent effects in reducing the CMV positivity rate. Thus, it is plausible to assume that the curve of positivity will stabilize around 17%, which is supported by the results of clinical trials indicating an approximately 40% decrease in positivity rate with the use of Letermovir compared with placebo group [20], already achieved at SU-PUI.

Finally, further reflection was provided by the analysis of the data pertaining to SOT category. In these patients, in fact, a redesign of the previously adopted post-transplant protocols proved to be sufficient in lowering the positivity rate of CMV in recent years. Thus, supporting the value of the road, already taken, directed to a personalized therapeutic approach, belying the strict necessity for novel drug discoveries.

### Electronic supplementary material

Below is the link to the electronic supplementary material.

Supplementary Material 1

## Data Availability

No datasets were generated or analysed during the current study.

## References

[1] Fishman JA (2007). Infection in solid-organ transplant recipients. N Engl J Med.

[2] Nowalk A, Green M (2016) Epstein-Barr Virus. 10.1128/microbiolspec.DMIH2-0011-2015. Microbiol Spectr 4 10.1128/microbiolspec.DMIH2-0011-2015 27337443

[3] Kotton CN (2013). CMV: Prevention, diagnosis and therapy. Am J Transpl.

[4] Al Hamed R, Bazarbachi AH, Mohty M (2020). Epstein-Barr virus-related post-transplant lymphoproliferative disease (EBV-PTLD) in the setting of allogeneic stem cell transplantation: a comprehensive review from pathogenesis to forthcoming treatment modalities. Bone Marrow Transpl.

[5] Bernard-Valnet R, Koralnik IJ, Du Pasquier R (2021). Advances in treatment of progressive multifocal leukoencephalopathy. Ann Neurol.

[6] Chong S, Antoni M, Macdonald A, Reeves M, Harber M, Magee CN (2019). BK virus: current understanding of pathogenicity and clinical disease in transplantation. Rev Med Virol.

[7] Liu BM, Hill HR (2020). Role of host immune and inflammatory responses in COVID-19 cases with underlying primary immunodeficiency: a review. J Interferon Cytokine Res.

[8] Candel FJ, Barreiro P, Salavert M, Cabello A, Fernández-Ruiz M, Pérez-Segura P (2023). Expert consensus: main risk factors for poor prognosis in COVID-19 and the implications for targeted measures against SARS-CoV-2. Viruses.

[9] Ljungman P, de la Camara R, Robin C, Crocchiolo R, Einsele H, Hill JA et al (2019) Guidelines for the management of cytomegalovirus infection in patients with haematological malignancies and after stem cell transplantation from the 2017 European Conference on Infections in Leukaemia (ECIL 7). Lancet Infect Dis 1;19:e260–72. 10.1016/S1473-3099(19)30107-0 10.1016/S1473-3099(19)30107-0 31153807

[10] Pretagostini R, Poli L, Lai Q, Russo G, Nudo F, Garofalo M (2017). Pre-emptive therapy for the treatment of Cytomegalovirus after kidney transplantation. Transpl Proc.

[11] Ling PD, Lednicky JA, Keitel WA, Poston DG, White ZS, Peng R (2003). The dynamics of herpesvirus and polyomavirus reactivation and shedding in healthy adults: a 14-month longitudinal study. J Infect Dis.

[12] Fuji S, Kapp M, Grigoleit GU, Einsele H (2011). Adoptive immunotherapy with virus-specific T cells. Best Pract Res Clin Haematol.

[13] Danziger-Isakov L, Blumberg EA, Manuel O, Sester M (2021). Impact of COVID-19 in solid organ transplant recipients. Am J Transpl.

[14] Szczawińska-Popłonyk A, Jończyk-Potoczna K, Ossowska L, Bręborowicz A, Bartkowska-Śniatkowska A, Wachowiak J (2014). Cytomegalovirus pneumonia as the first manifestation of severe combined immunodeficiency. Cent Eur J Immunol.

[15] Di Mattia G, Nenna R, Mancino E, Rizzo V, Pierangeli A, Villani A (2021). During the COVID-19 pandemic where has respiratory syncytial virus gone?. Pediatr Pulmonol.

[16] Olsen SJ, Azziz-Baumgartner E, Budd AP, Brammer L, Sullivan S, Pineda RF (2020). Decreased influenza activity during the COVID-19 pandemic - United States, Australia, Chile, and South Africa, 2020. MMWR Morb Mortal Wkly Rep.

[17] Martino M, Pitino A, Gori M, Bruno B, Crescimanno A, Federico V (2021). Letermovir Prophylaxis for Cytomegalovirus infection in allogeneic stem cell transplantation: a real-world experience. Front Oncol.

[18] Gaur N, Dharwadkar R, Thomas J, Malviya R, Ghinea G, Danaraj RK, Balusamy B, Sundram S (2022). Personalized therapy using deep learning advances. Deep learning for targeted treatments.

[19] Visser E, Milne D, Collacott I, McLernon D, Counsell C, Vickers M (2014). The epidemiology of infectious Mononucleosis in Northern Scotland: a decreasing incidence and winter peak. BMC Infect Dis.

[20] El Helou G, Razonable RR (2019). Letermovir for the prevention of cytomegalovirus infection and disease in transplant recipients: an evidence-based review. Infect Drug Resist.

