# Supplementary material for: Molecular monitoring of viral infections in immunocompromised patients in a large university hospital in Italy: reflections after thirteen years of real-life activity
Source: Eur J Clin Microbiol Infect Dis. 2024 Mar 22;43(5):979–89. doi: 10.1007/s10096-024-04812-z (PMC11108949; doi:10.1007/s10096-024-04812-z)
Supplement: Supplementary file 1 — Supplementary Material 1 [file 10096_2024_4812_MOESM1_ESM.docx]

| **Table S1.** | Number of requests and positivity rate for the different biological samples tested for BKV and JCV viruses. | | | | | | |
| --- | --- | --- | --- | --- | --- | --- | --- |
|  |  | **JCV** | | | **BKV** | | |
|  |  | Plasma | Urine | CSF | Plasma | Urine | CSF |
| Sample | Total | 1666 | 786 | 2199 | 2924 | 1058 | 2025 |
|  | Positive | 202 | 288 | 46 | 457 | 425 | 32 |
|  | Positivity (%) | 12 | 36.6 | 2.1 | 15.6 | 40 | 1.6 |
